# Reduced ribosomal DNA transcription in the prefrontal cortex of suicide victims: consistence of new molecular RT-qPCR findings with previous morphometric data from AgNOR-stained pyramidal neurons

**DOI:** 10.1007/s00406-021-01232-4

**Published:** 2021-01-26

**Authors:** Marta Krzyżanowska, Krzysztof Rębała, Johann Steiner, Michał Kaliszan, Dorota Pieśniak, Karol Karnecki, Marek Wiergowski, Ralf Brisch, Katharina Braun, Zbigniew Jankowski, Monika Kosmowska, Joanna Chociej, Tomasz Gos

**Affiliations:** 1 Department of Forensic Medicine, Medical University of Gdańsk, ul. Dębowa 23, 80-204 Gdańsk, Poland; 2 Department of Psychiatry, Otto von Guericke University, Magdeburg, Germany; 3 Department of Zoology/Developmental Neurobiology, Institute of Biology, Otto von Guericke University, Magdeburg, Germany

**Keywords:** Postmortem, Suicide, Prefrontal cortex, RT-qPCR

## Abstract

Prefrontal cortical regions play a key role in behavioural regulation, which is profoundly disturbed in suicide. The study was carried out on frozen cortical samples from the anterior cingulate cortex (dorsal and ventral parts, ACd and ACv), the orbitofrontal cortex (OFC), and the dorsolateral cortex (DLC) obtained from 20 suicide completers (predominantly violent) with unknown psychiatric diagnosis and 21 non-suicidal controls. The relative level of ribosomal RNA (rRNA) as a marker of the transcriptional activity of ribosomal DNA (rDNA) was evaluated bilaterally in prefrontal regions mentioned above (i.e. in eight regions of interest, ROIs) by reverse transcription and quantitative polymerase chain reaction (RT-qPCR). The overall statistical analysis revealed a decrease in rDNA activity in suicide victims versus controls, particularly in male subjects. Further ROI-specific *post hoc* analyses revealed a significant decrease in this activity in suicides compared to non-suicides in five ROIs. This effect was accentuated in the ACv, where it was observed bilaterally. Our findings suggest that decreased rDNA transcription in the prefrontal cortex plays an important role in suicide pathogenesis and corresponds with our previous morphometric analyses of AgNOR-stained neurons.

**Supplementary Information:**

The online version contains supplementary material available at 10.1007/s00406-021-01232-4.

## Introduction

Prefrontal cortex (PFC) regions play a key role in behavioural regulation, which is profoundly disturbed in suicide victims. Impaired executive functions with poor impulse control, problem-solving and decision-making are the outstanding manifestations of PFC dysfunction preceding suicidal behaviour (for reviews see: [1, 2]). Numerous neurobiological data are suggestive of the distinctness of suicide in mental disorders (for reviews see: [1, 2]), which has been considered in the fifth edition of the Diagnostic and Statistical Manual of Mental Disorders—DSM V [3]. Neuropathological studies of our workgroup have also suggested this distinctness [4–7] (for a review of our previous studies see: [8]).

The transcription of ribosomal genes is crucial for key cellular functions in the brain, *inter alia*, the outgrowth of neurites [9–11] and myelination process [12], both deteriorated in the PFC of suicide victims ([13] and [14], respectively). Disturbed ribosomal function in the PFC in suicide has been highlighted in recent exploratory microarray analysis of genetic variants in suicide completers [15]. As revealed by molecular research, rDNA transcription is attenuated in the hippocampus in cases with a history of child abuse and this effect is related to epigenetic modulations [16], which are also observed in the PFC [17, 18]. Similar results were obtained in the animal model of early-life stress [19], which is one of well-established and strongest distal factors in stress-diathesis model of suicidal behaviour [1, 2].

As rDNA transcription is affected by multiple factors [20], there are numerous molecular signatures involved in the dysregulation of this key cellular process in suicide, which have been revealed recently. Besides epigenetic phenomena, current molecular suicide research points to disturbances of stress axis components [21, 22] (for a review see: [23]), malfunction of trophic factors [21] (for a review see: [24]), glutamatergic system diathesis [25–27], abnormal glial–neuronal interaction [14, 28–31] and cross-talk between immune system and brain [32, 33].

Our previous morphological studies by the AgNOR staining method have suggested the attenuated rDNA transcription in prefrontal pyramidal neurons as a phenomenon specific for suicidal patients with established diagnoses of unipolar or bipolar depression [34, 35]. Our recent AgNOR study has suggested the same effect in forensic cohort of suicide victims with unknown psychiatric diagnosis [4]. Silver-stained nucleolar organising regions (AgNORs) clustered together in the nucleolus in the AgNOR area, which is observed by light microscopy, represent the site of rDNA transcription in human interphase cells [4, 34, 35]. The transcriptional activity of rDNA can be assessed by measuring AgNOR parameters, among them the AgNOR area, which is decreased in prefrontal pyramidal neurons of suicide completers [4].

The distinctness of suicide-specific abnormalities in the PFC has been supported by a study on microglial reaction from a workgroup in Magdeburg, which was found to be increased in suicidal patients from different groups of mental disorders [28]. This effect has been verified by studies of other workgroups [29, 30]. The oxidative stress as a deleterious consequence of microglia hyperactivity in turn may lead to the decreased rDNA transcription in affected cellular elements of the PFC [12, 36, 37].

Therefore, in the present study, we hypothesized a decreased rDNA transcriptional activity in prefrontal regions of suicide completers regardless of their underlying psychiatric diagnosis (i.e. independent of psychiatric comorbidity) and tested this hypothesis by the application of reverse transcription and quantitative polymerase chain reaction (RT-qPCR) in forensic postmortem material. We aimed at both basic research on the neurobiology of suicide and the informative comparison between molecular and morphological evaluation of rDNA transcription in the same pool of brain samples [4], which has not been reported previously.

## Materials and methods

### Human brain tissue

Prefrontal parts of both hemispheres of 20 suicide victims (14 males/6 females) with unknown data both on psychiatric comorbidity and on possible psychotropic medication preceding death (typical for most of suicide cases autopsied in our Department of Forensic Medicine) and 21 (17 males/4 females) controls were obtained during routine forensic autopsies in accordance with existing EU law regulations. The study has been approved by the local ethics committee of the Medical University of Gdańsk as performed in accordance with the ethical standards laid down in the Declaration of Helsinki of 1989.

The detailed diagnostic and demographic data of investigated cases are present in the Supplementary Table. Violent suicide methods prevailed in the suicide cohort (13 out of 20), which is representative for our autopsy material. Control cases of unnatural manner of death were more numerous than those of natural manner (13 and 8, respectively). Only sudden death cases were investigated in suicide and control cohorts. All brains were free of gross neuropathology suggestive of vascular, traumatic, inflammatory, neoplastic and neurodegenerative processes. Macroscopic evaluation of brains was confirmed by histopathological investigation in cases, where the cause of death was unclear at autopsy and the routine histopathological evaluation of internal organs was necessary for the forensic diagnosis (i.e. in seven non-violent suicide cases of self-poisoning by medication overdose and eight control cases of natural manner of death). Neither chronic nor acute pathological processes were observed microscopically in these cases in neocortical areas and other brain regions in hematoxylin–eosin-stained sections. Among others, neuronal necrosis as a consequence of protracted antemortem hypoxia was excluded by histopathological investigation. Neurodegenerative changes, such as amyloid plaques, perivascular amyloid deposits and neurofibrillary tangles, were not observed microscopically in the AgNOR staining in prefrontal regions in the same cohorts investigated previously [4]. Blood and urine were tested for the presence of ethanol at each autopsy. The majority of investigated cases (17 suicide victims and 15 controls) revealed the blood alcohol concentration (BAC) below the limit of quantification (LOQ), i.e. < 0.2 g/l according to internationally accepted analytical guidelines. The remaining three suicide victims and 6 controls revealed BAC in the range of 0.59–3.4 g/l (the highest value in a case of self-poisoning by quetiapine) and 0.74–3.15 g/l (the highest value in one of stabbed cases), respectively. Other substances of abuse, antidepressant and antipsychotic drugs, as well as their metabolites were investigated when intoxication was suggested by the scene inspection and/or other available information sources prior to the autopsy, i.e. in seven cases. These cases constituted the non-violent suicide subgroup.

**Fig.1:**
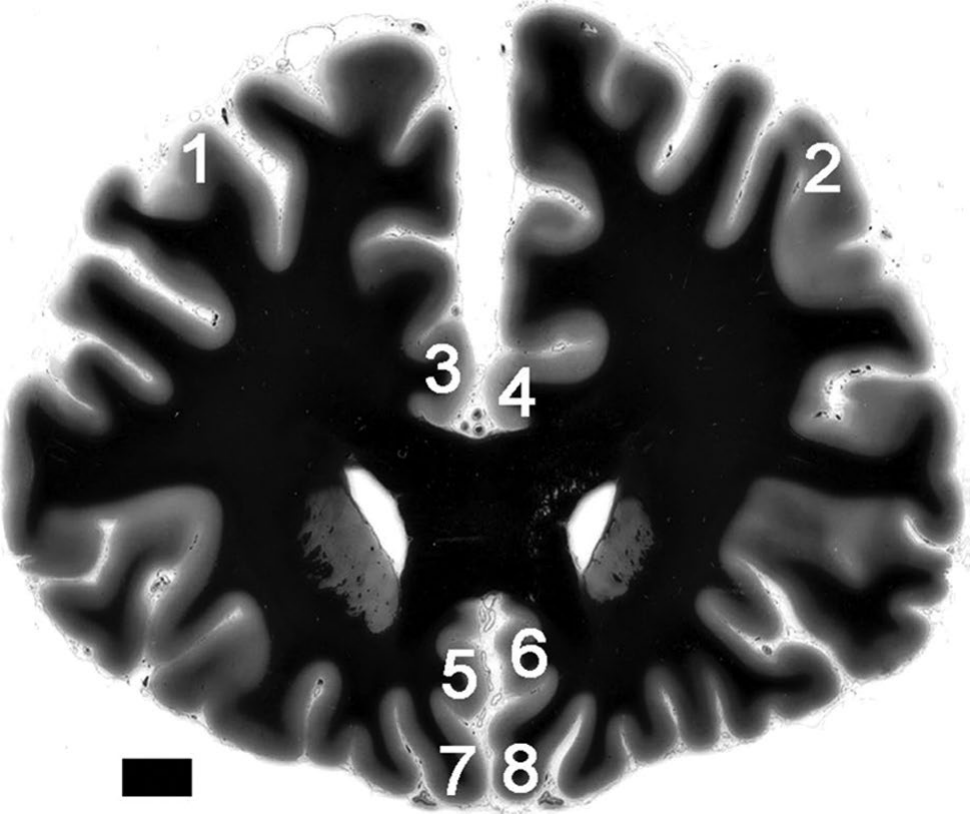
Regions of interest in the prefrontal cortex are shown bilaterally (1—dorsolateral right, 2—dorsolateral left; 3—anterior cingulate dorsal right, 4—anterior cingulate dorsal left; 5—anterior cingulate ventral right, 6—anterior cingulate ventral left; 7—orbitofrontal right, 8—orbitofrontal left) at low magnification picture of the Nissl-myelin-stained coronal section at the level where investigations were carried out (scale bar 10 mm; the staining method is described in [67])

Prefrontal parts of the brains were separated at forensic autopsies from both hemispheres by coronal sections at the level of temporal poles. Immediately after the separation, cortical samples for the molecular rRNA assays were isolated closely to the section plane from the following prefrontal regions: dorsolateral prefrontal cortex (DLC), anterior cingulate cortex (dorsal (ACd) and ventral (ACv) parts) and orbitofrontal cortex (OFC) (see Fig. 1). After isolation of the samples, the remaining prefrontal parts were fixed for the morphological investigations of the same cortical regions by the AgNOR staining method, which were presented previously [4].

### rRNA isolation and reverse transcription

Immediately after the separation from the brain, cortical samples were vortexed, double-centrifuged (60 s, 5 000 RCF, room temperature) and incubated overnight at 4–8 °C in RNA-stabilising RNA*later* Solution (Thermo Fisher Scientific, USA). Subsequently, they were stored in this solution at – 54 °C until RNA extraction. All samples were blinded by pseudonymization through assigning consecutive case numbers regardless of the forensic diagnosis of suicide vs. non-suicide.

Total RNA present in the frozen samples was extracted with a GeneMATRIX Universal DNA/RNA/Protein Purification Kit (EURx, Poland) with an additional step of treatment of RNA isolate with desoxyribonuclease I (DNAse I). RNA concentration was measured spectrophotometrically in a NanoDrop ND-1000 UV–Vis spectrophotometer (NanoDrop Technologies, USA). RNA (100 ng) was reversely transcribed with a TranScriba Kit (A&A Biotechnology, Poland) with an RNase inhibitor and random hexamer oligonucleotides as primers according the producer’s instructions. As opposed to oligo(dT) primers commonly used in routine reverse transcription, random hexanucleotides enable cDNA synthesis for rRNA, which is not stabilised in the cell via polyadenylation. Final cDNA concentration of 5 ng/µl after the reverse transcription step was sufficient for quantitative PCR.

### Real-time quantitative polymerase chain reaction

Fragments of *RNA18S5* (RNA, 18S ribosomal 5) as a target gene and *GAPDH* (glyceraldehyde 3-phosphate dehydrogenase) as a reference gene were amplified simultaneously in one 5 µL reaction by duplex qPCR in a 7900HT Fast Real-Time PCR System instrument (Applied Biosystems, USA) with the use of TaqMan Gene Expression Master Mix (Thermo Fisher Scientific) and predesigned TaqMan Gene Expression Assays: Hs03928990 with a FAM-labelled TaqMan probe specific for *RNA18S5*, and Hs03929097 with a VIC-labelled TaqMan probe specific for *GAPDH* (Thermo Fisher Scientific). The housekeeping gene *GAPDH* was proven to be one of the most stable reference genes [38] used for normalisation of gene expression data also in current molecular suicide research on prefrontal samples [39, 40]. All prefrontal samples and non-template contamination controls were analysed in four replicates.

Amplification was performed in standard mode thermal cycling conditions with initial 2-min uracil-DNA glycosylase (UDG) incubation (50 °C) for prevention of carry-over PCR contamination and with 10-min AmpliTaq Gold DNA polymerase activation (95 °C). Due to the late signal of amplification of the reference *GAPDH* gene (resulting from the high cellular expression level of rDNA), real-time amplification data were collected for as many as 80 qPCR cycles. PCR parameters were set according to the manufacturer’s instructions: 15 s of denaturation at 95 °C followed by 1 min of annealing and elongation at 60 °C.

### Data analysis

The relative expression level of rDNA was estimated by comparison of the expression level of *RNA18S5* to the expression level of *GAPDH* from values of a threshold cycle (C_T_) in which fluorescence of fluorophore released during qPCR exceeded the background level. The analysis was performed with the use of SDS v2.2 software (Applied Biosystems) with automatic setting of the baseline and threshold values for the determination of C_T_ from amplification curves.

Statistical analyses were performed with the data analysis software system STATISTICA version 10 (StatSoft®, Inc. 2011, www.statsoft.com). As normal distribution was not given for analysed data (i.e. significant values of Kolmogorov–Smirnov tests were obtained), non-parametric statistical procedures were used in hierarchic mode.

First, STATISTICA generalized linear/nonlinear models (GLZ) module containing general custom designs (GCD) procedure was applied as an omnibus method to analyse associations between dependent variable, i.e. the relative rRNA level and independent categorical variables (i.e. suicidal/control group, PFC region, cortical layer, and sex as the categorical confounding variable). The results of the GCD analyses were reported automatically including the Wald statistic value, degrees of freedom, and the respective *P* value.

Age, postmortem interval, brain weight and BAC (values below LOQ were accounted null values in statistical analysis) were considered as numerical confounding variables. Therefore, the subsequent GCD procedure was applied in each of ROIs to analyse associations between the relative rRNA level and these variables. Supplementary to GCD analyses in ROIs, Spearman’s correlation coefficients were calculated to determine the impact of numerical variables which might confound the dependent variable. These coefficients were also calculated to determine correlations between rRNA levels and AgNOR area values in prefrontal pyramidal neurons found previously [4].

Following the GCD analyses, unadjusted two-way post hoc comparisons with Mann–Whitney *U* test and the *χ*^*2*^ test were used to detect the possible differences between the studied groups with respect to the variables mentioned above (i.e. rRNA level and confounders). All statistical tests were two-tailed. In general, *P* values of < 0.05 were accepted as statistically significant.

Kruskal–Wallis analysis of the variance of ranks (*H* test) with subsequent *U* tests was performed for the evaluation of differences in the rRNA level related to sex between suicides and controls, which was suggested by the GCD analysis; in this procedure, *U* test *P* values were adjusted to multiple comparisons according to the Bonferroni correction. The same procedure was applied for the evaluation of differences in dependent variable between subgroups of suicide victims (violent vs. non-violent) and controls (unnatural vs. natural manner of death).

## Results

### The analysis of rRNA relative levels

Cumulative analysis of results from all 8 investigated ROIs (i.e. from all cortical samples obtained from 4 evaluated PFC regions bilaterally, 159 suicidal and 167 control values) by GCD procedure revealed the association of forensic diagnosis (i.e. suicide vs. non-suicide) with the rRNA relative level (Wald statistic = 14.951, df = 1, *P* = 0.000110, median values 6.309 and 9.153 for suicides and non-suicidal controls, respectively, see Table 1). The effect of diagnosis was not associated with the brain hemisphere or investigated PFC region. Instead, it was associated with sex, i.e. mainly driven by male subjects (see Tables 1 and 2, and the next paragraph).

**Table 1:** Statistical data regarding the intergroup comparisons of rRNA relative levels between controls (*n* = 21) and suicide victims (*n* = 20): the presentation of general analyses of results

	Sex	Age (yr)	PMI (h)	BAC (g/l)	rRNA all (m + f)	rRNA all (m)	rRNA all (f)
Controls							
Ratio/median (q1, q3)	17 m/4f	56 (25, 61)	24 (12, 30)	0.00 (0.00, 0.59)	9.153 (5.960, 14.456)	9.809 (6.026, 15.146)	7.444 (5.174, 10.018)
Suicide victims							
Ratio/median (q1, q3)	14 m/6f	45 (35.5, 58.5)	24 (24, 30)	0.00 (0.00, 0.00)	6.309 (4.542, 9.102)	6.458 (5.004, 9.102)	4.843 (2.657, 8.757)
Statistics							
Test	*χ*^*2*^ test	*U*	*U*	*U*	GCD	GCD	GCD
Characteristic value	*χ*^*2*^ = 0.670	*Z* = − 0.157	*Z* = − 0.612	*Z* = 1.062	Wald statistic = 14.951	Wald statistic = 12.537	Wald statistic = 1.038
*P* value	0.414	0.867	0.561	0.446	**0.000110**	**0.000399**	0.308

Significant *P* values are in bold

*PMI* postmortem interval, *BAC* blood alcohol concentration; all (m + f) —cumulated results from all analysed regions of interest in both sexes, all (m)—in males, all (f)—in females; *m* males, *f* females, *q1* and *q3* quartile 1 and 3, *GCD* general custom designs procedure (in generalized linear/nonlinear models of data analysis software system STATISTICA)

**Table 2:** The presentation of intergroup comparisons between rRNA relative levels in regions of interest, taking into account the data from both sexes, and separately the data from males and females

	ACd right	ACd left	ACv right	ACv left	OFC right	OFC left	DLC right	DLC left
Controls								
Median (q1, q3)								
Males and females (*n* = 21)	8.774 (6.329, 15.962)	10.312 (6.058, 17.497)	10.137 (6.254, 12.399)	8.287 (5.557, 15.011)	8.509 (5.995, 15.552)	8.737 (5.626, 14.015)	9.477 (5.960, 14.015)	8.170 (5.605, 11.679)
Males (*n* = 17)	11.901 (6.824, 21.415)	11.282 (6.058, 20.569)	10.872 (6.617, 13.560)	9.522 (5.557, 15.936)	8.509 (6.634, 15.552)	9.444 (6.665, 14.015)	9.477 (5.667, 14.015)	8.170 (5.605, 12.115)
Females (*n* = 4)	6.959 (5.568, 8.218)	9.056 (6.251, 10.753)	6.342 (4.292, 8.667)	7.744 (6.282, 9.557)	7.924 (4.846, 15.754)	6.885 (4.895, 16.766)	9.844 (7.557, 14.845)	8.382 (5.002, 9.601)
Suicide victims								
Median (q1, q3)								
Males and females (*n* = 20)	7.105 (4.738, 10.729)	6.864 (4.843, 8.174)	6.337 (4.146, 7.854)	5.152 (3.752, 9.963)	6.016 (3.969, 9.197)	7.555 (5.898, 9.418)	6.214 (4.052, 7.409)	5.201 (3.359, 8.230)
Males (*n* = 14)	8.096 (5.273, 10.599)	7.149 (6.097, 8.331)	6.963 (4.990, 7.854)	5.152 (4.803, 10.204)	6.016 (4.734, 8.893)	6.722 (5.993, 10.904)	6.338 (5.909, 7.591)	5.773 (4.719, 8.134)
Females (*n* = 6)	5.425 (3.499, 15.622)	4.843 (2.140, 6.994)	5.469 (2.401, 7.387)	3.923 (2.318, 7.043)	5.350 (2.834, 10.648)	8.712 (2.798, 9.251)	3.854 (2.067, 6.270)	3.262 (2.203, 8.327)
Statistics								
*U* test* P* values*								
Males and females	0.122	**0.020**	**0.019**	**0.048**	**0.015**	0.249	**0.022**	0.122
Males	0.104	0.068	**0.014**	0.124	**0.019**	0.249	0.190	0.234
Females	0.669	0.201	0.522	0.201	0.394	1.000	0.055	0.394

Significant *P* values are in bold

*ACd* anterior cingulate cortex, dorsal part, *ACv* anterior cingulate cortex, ventral part, *OFC* orbitofrontal cortex, *DLC* dorsolateral prefrontal cortex, *q1* and *q3* quartile 1 and 3, *n* number of individuals, * corrected for multiple comparisons by the Bonferroni procedure

Further ROI-specific analyses by *U* tests revealed rRNA decrease in suicides compared to non-suicides in all eight ROIs. This effect was significant in five of them and was accentuated in the ACv, where it was observed bilaterally (see Table 2).

No significant differences existed in the rRNA level between violent (*n* = 13) and non-violent (*n* = 7) suicide victims as well as between control cases of unnatural (*n* = 13) and natural manner of death (*n* = 8) in any of ROIs (non-significant *U* test *P* values corrected for multiple comparisons).

### Confounders

Suicidal and control groups were matched by gender (non-significant *χ*^*2*^ test *P* value, Table 1 and Supplementary Table) and no significant intra-group differences between sexes were found in the rRNA relative level in any of analysed ROIs (non-significant *U* test *P* values corrected for multiple comparisons). However, according to the associated effect of diagnosis and sex suggested by the initial GCD procedure, only males revealed an association between forensic diagnosis and rRNA level in the cumulative analysis of all investigated ROIs (see Table 1). Following this initial evaluation, only male suicide victims revealed significant decreases in the rRNA level compared to male controls in ROI-specific analyses (significant *U* test *P* values corrected for multiple comparisons in the right ACv and OFC, see Table 2). Therefore, the observed phenomenon was specific for males.

Age and PMI revealed no significant differences between suicides and controls (non-significant *U* test *P* values, Table 1 and Supplementary Table), similar to brain weight and BAC. ROI-specific analyses by GCD procedure revealed no associated impact of any of those numerical confounders and forensic diagnosis on rRNA levels (non-significant Wald statistic *P* values) in any of investigated ROIs. Correspondingly, no relevant correlations were found between numerical confounders and rRNA level in any ROI in both groups (non-significant Spearman’s correlation *P* values and/or irrelevant *r* values). Therefore, the observed differences in the rRNA level between suicides and controls both in the cumulative and ROI-specific analyses were not confounded by these variables.

### Correlation with previous AgNOR findings

An additional correlation analysis was performed between rRNA levels and mean values of AgNOR area calculated from the values of this parameter in prefrontal pyramidal neurons in layers III and IV, which were obtained previously [4]. This analysis revealed significant negative correlations in control group, whereas no correlations between current molecular and previous morphological results were found in any ROI in suicide victims (non-significant Spearman’s correlation *P* values and irrelevant *r* values, see Table 3).

**Table 3:** Regions of interest-specific correlation analysis between rRNA relative levels and AgNOR area mean values in pyramidal neurons

	ACd right	ACd left	ACv right	ACv left	OFC right	OFC left	DLC right	DLC left
Controls r/P	** − 0.51/0.016**	** − 0.54/0.010**	− 0.30/0.180	** − 0.46/0.038**	** − 0.53/0.011**	− 0.30/0.182	− 0.36/0.105	− 0.16/0.490
Suicides r/P	− 0.17/0.448	− 0.06/0.808	− 0.03/0.885	0.003/0.988	− 0.10/0.654	− 0.12/0.594	− 0.28/0.213	− 0.15/0.505

Significant values are in bold

*r *correlation coefficient, *P* *P* value of the Spearman’s correlation, *ACd* anterior cingulate cortex, dorsal part, *ACv* anterior cingulate cortex, ventral part, *OFC* orbitofrontal cortex, *DLC* dorsolateral prefrontal cortex

## Discussion

We have found the decreased rDNA transcription in PFC regions in suicide victims, which was specific for male suicides, similarly to our previous study of prefrontal pyramidal neurons by the AgNOR staining method [4]. There are different possible interpretations of this finding. Experimental studies in animal models of chronic stress, which is an established proximal factor in suicide [1, 2], revealed in prefrontal pyramidal neurons sex-specific differences in the formation of dendrites (for a review see: [41]), i.e. in the process closely related to rDNA activity [9–11]. Correspondingly, different expression of genes related to the formation of neuronal processes has been observed in the PFC of male compared to female suicide completers [42, 43]. However, the small sample size of female subjects may lead to an underpowered analysis and there is a chance of false negative results. Therefore, the results should be replicated in larger cohorts with more numerous female samples. Other variables which may confound present results, among them postmortem interval, did not influence our current results. The observed accentuated intra-group differences in rDNA expression profiles in bulk cortical tissue may be a consequence of differences in cellular composition related to the variation in gray/white matter ratios in the extracted tissue samples and/or inter-subject variability [44]. Our method does not allow to explain these differences.

The rDNA transcription was globally decreased in suicide in analysed prefrontal regions, which was accentuated in the cumulative evaluation of all ROIs simultaneously (see Table 1) and this effect was associated with neither the hemisphere nor the prefrontal region. This finding could be explained by close reciprocal connectivity between PFC areas which constitute a functional syncytium [45]. However, further region-specific *post hoc* analyses revealed significant bilateral decrease in ribosomal gene expression only in the ACv (see Table 1), i.e. in prefrontal region crucial for behavioural regulation [46]. Therefore, both our current molecular findings and previous morphological reports [4, 34, 35, 47] are in line with the aberrant regulatory function of the ventromedial PFC accentuated in suicidal behaviour [1, 2].

Our previous AgNOR studies of cohorts with both established diagnoses of affective disorders [34, 35] and unknown psychiatric comorbidity [4] suggested a decreased rDNA transcription in prefrontal pyramidal neurons as a phenomenon specific for suicide completers and our current results correspond with them. However, we have not found positive correlations between AgNOR area in these neurons and rRNA levels in samples of bulk cortical tissue in both controls and suicide victims. There are some possible explanations of this discrepancy. Pyramidal neurons constitute roughly 30% of prefrontal cells, and layer III and V neurons investigated in our study [4] are only part of them, whereas other cellular populations in the PFC include inhibitory interneurons (10%), oligodendrocytes (45%), astrocytes (12%) and microglia (3%) [48, 49]. Thus, abnormalities in other cell types besides pyramidal neurons, predominantly in oligodendrocytes, may also contribute to the decrease of rRNA synthesis in bulk PFC tissue observed in suicide victims. This hypothesis corresponds with previous morphological reports on cohorts including suicides with established diagnoses of affective disorders or schizophrenia, where decreased numbers of oligodendrocytes were found in the PFC [50, 51]. Interestingly, negative correlations between AgNOR area and rRNA level were found in controls, whereas they were lacking in suicide victims (see Table 3). The observed effect could suggest a reciprocal regulation of rRNA synthesis in prefrontal pyramidal neurons and oligodendrocytes in normal human brain. According to recent research, a molecular cross-talk exists between these cell types (for a review see: [52]). However, further molecular analysis of distinct cellular populations in the PFC is needed to explain the relation between rDNA transcriptional activity in pyramidal neurons and oligodendrocytes.

The silver staining in AgNOR areas in interphase cells is related predominantly to the multifunctional protein nucleolin (important for the function of RNA-polymerase I) but not directly to the rRNA amount [53, 54]. This could be a further possible explanation of the discrepancy between our current molecular and previous morphological study. As numerous factors are involved in the regulation of rRNA synthesis crucial for cellular functions [20], the amount of nucleolin may not correlate directly with the level of rDNA transcription. On the one hand, molecular suicide research suggests that rDNA transcription in the PFC may be downregulated more directly by the hypermethylation of rDNA promoter region, i.e. by the epigenetic phenomenon [17, 18]. On the other hand, however, the key node in the intracellular regulation of rDNA transcription is the mammalian target to rapamycin (mTOR) [55], which is disturbed in the PFC in suicide [56]. Moreover, suicide research revealed abnormalities of different molecular factors related to the function of mTOR.

Among the most important are molecular components of stress axis, which is profoundly disturbed in suicidal behaviour [1, 2]. Both corticotropin-releasing hormone receptor 1 [22] and glucocorticoid receptor (for a review see: [23]) are decreased in the PFC in suicide, which may impact rDNA transcription [57–59]. The abnormalities in molecules regulating glucocorticoid receptor function, which have been found in suicide [21, 60, 61], may also contribute to the attenuated rRNA synthesis in prefrontal pyramidal cells and oligodendrocytes [21].

The stress axis function is closely related to glutamatergic neurotransmission [57], which is disturbed in suicidal behaviour in the PFC [25–27]. Impaired function of brain-derived neurotrophic factor (BDNF) is an important molecular effect of this disturbed neurotransmission [62]. The decrease of BDNF has been found in prefrontal regions of suicide victims (for a review see: [24]), which may inhibit rDNA transcription in neurons [62] and oligodendrocytes [12].

Besides glucocorticoids, glutamatergic neurotransmission is profoundly influenced by activated microglia producing pro-inflammatory cytokines [63]. Both microglial reaction [28–30] and these cytokine levels [32, 33] are increased in the PFC of suicide victims. Subsequent oxidative stress may attenuate rRNA synthesis in pyramidal neurons [64, 65] and oligodendrocytes [12, 66] leading to disturbed synaptic formation [65] and deteriorated myelin production [14], respectively.

## Limitations

The present study has certain limitations that have to be considered: (1) A relatively small number of predominantly male cases was analyzed. Therefore, results have to be confirmed in a larger sample with more numerous female subjects. (2) The psychiatric diagnoses (also including substance use disorders) and the data on possible psychotropic medication preceding death were not available. The levels of psychotropic drugs were established only in seven suicide victims where medication overdose constituted a cause of death. However, our current study did not aim at analysis of relation between suicide and other mental disorders and our previous studies did not suggest that the decreased rDNA activity in prefrontal pyramidal neurons may be related to the medication used in the last three months of life [13, 15]. (3) As we used bulk tissue homogenates, our method does not allow to differentiate between cell types, in which the observed phenomenon of decreased rDNA transcription occurs. (4) Our relative rDNA expression estimates are based on assumption of stable housekeeping *GAPDH* gene expression across subjects and samples.

## Conclusion

In summary, our results suggest decreased rDNA transcription in the PFC in male suicide victims as a presumable consequence of multiple molecular events. The molecular results correspond with our previous morphological research on PFC pyramidal neurons in suicide based on the AgNOR staining method.

## Supplementary Information

Below is the link to the electronic supplementary material.

Supplementary file1 (DOCX 34 KB)

## Data Availability

On behalf of all authors, the corresponding author states that the data being reported are accurate and are coming from the official source.
