# Supplementary material for: Reduced ribosomal DNA transcription in the prefrontal cortex of suicide victims: consistence of new molecular RT-qPCR findings with previous morphometric data from AgNOR-stained pyramidal neurons
Source: Eur Arch Psychiatry Clin Neurosci. 2021 Jan 26;271(3):567–76. doi: 10.1007/s00406-021-01232-4 (PMC7981327; doi:10.1007/s00406-021-01232-4)
Supplement: Supplementary file 1 — Supplementary file1 (DOCX 34 KB) [file 406_2021_1232_MOESM1_ESM.docx]

**Reduced ribosomal DNA transcription in the prefrontal cortex of suicide victims: Consistence of new molecular RT-qPCR findings with previous morphometric data from AgNOR-stained pyramidal neurons**

Marta Krzyżanowska, Krzysztof Rębała, Johann Steiner, Michał Kaliszan, Dorota Pieśniak, Karol Karnecki, Marek Wiergowski, Ralf Brisch, Katharina Braun, Zbigniew Jankowski, Monika Kosmowska, Joanna Chociej, Tomasz Gos

**European Archives of Psychiatry and Clinical Neuroscience**

Corresponding author:

Tomasz Gos, MD, PhD

Department of Forensic Medicine

Medical University of Gdańsk

ul. Dębowa 23

80-204 Gdańsk, Poland

E-mail: [gost@gumed.edu.pl](mailto:gost@gumed.edu.pl)

**Supplementary Table** Detailed diagnostic and demographic data, and the values of rRNA relative levels in regions of interest in controls (n=21) and suicide victims (n=20). *Abbreviations:* ACd – anterior cingulate cortex, dorsal part; ACv – anterior cingulate cortex, ventral part; f – female; m – male; PMI – postmortem interval; BAC – blood alcohol concentration; LOQ – limit of quantification (BAC = 0.2 g/l); *q1* and *q3* – quartile 1 and 3.

| Case ID | Cause of death | Sex | Age | PMI | BAC | ACd right | ACd left | ACv right | ACv left |
| --- | --- | --- | --- | --- | --- | --- | --- | --- | --- |
|  | **Controls** |  |  |  |  |  |  |  |  |
| 1 | Sudden cardiac death | m | 61 | 24 | <LOQ | 8.919 | 10.152 | 14.456 | 3.755 |
| 2 | Traffic accident (motorcycle rider, injury of subclavian artery and traumatic haemothorax) | m | 25 | 12 | <LOQ | 5.270 | 9.341 | 7.430 | 5.491 |
| 3 | Right ventricular failure | m | 58 | 48 | <LOQ | 3.044 | 3.769 | 6.254 | 4.045 |
| 4 | Sudden cardiac death | m | 63 | 15 | <LOQ | 5.939 | 6.058 | 4.459 | 4.018 |
| 5 | Traffic accident (pedestrian injured in collision with motorcycle, multiple injuries) | m | 24 | 12 | <LOQ | 28.262 | 47.470 | 58.225 | 58.639 |
| 6 | Pulmonary embolism (acute cor pulmonale) | f | 59 | 24 | <LOQ | 4.880 | 11.195 | 3.563 | 4.876 |
| 7 | Traffic accident (car occupant, multiple injuries) | m | 20 | 24 | <LOQ | 6.329 | 5.605 | 5.764 | 6.561 |
| 8 | Traffic accident (car occupant, multiple injuries) | f | 59 | 24 | <LOQ | 6.256 | 4.703 | 5.022 | 7.688 |
| 9 | Traffic accident (pedestrian injured in collision with motorcycle, multiple injuries) | m | 24 | 12 | 0.80 | 6.991 | 5.128 | 6.617 | 5.623 |
| 10 | Sudden cardiac death | m | 45 | 12 | <LOQ | 11.901 | 8.174 | 10.137 | 6.132 |
| 11 | Stabbed (open wounds of thorax) | m | 57 | 10 | 1.70 | 54.348 | 26.802 | 47.302 | 47.883 |
| 12 | Transport accident (pedestrian injured in collision with railway vehicle, injury of cervical spinal cord) | f | 19 | 20 | <LOQ | 7.662 | 7.800 | 7.662 | 7.800 |
| 13 | Stabbed (open wound of thorax) | m | 28 | 7 | 3.15 | 6.824 | 5.991 | 5.645 | - |
| 14 | Traffic accident (pedestrian injured in collision with car, multiple injuries) | m | 71 | 24 | 0.74 | 21.415 | 20.569 | 10.907 | 11.470 |
| 15 | Traffic accident (car driver, multiple injuries) | m | 32 | 30 | 0.59 | 48.495 | 43.965 | 12.399 | 20.310 |
| 16 | Work-related transport accident (pedestrian injured in collision with railway vehicle, multiple injuries) | m | 56 | 48 | <LOQ | 15.962 | 26.100 | 14.491 | 10.269 |
| 17 | Traffic accident (pedestrian injured in collision with car, multiple injuries) | m | 66 | 24 | <LOQ | 26.100 | 13.754 | 10.269 | 15.962 |
| 18 | Diabetic coma with ketoacidosis | m | 39 | 48 | <LOQ | 12.623 | 11.854 | 13.560 | 15.909 |
| 19 | Sudden cardiac death | m | 64 | 48 | <LOQ | 15.909 | 11.282 | 10.872 | 14.112 |
| 20 | Work-related injury of head | m | 21 | 64 | 1.07 | 8.401 | 17.497 | 11.314 | 8.774 |
| 21 | Accidental fall from building | f | 72 | 15 | <LOQ | 8.774 | 10.312 | 9.673 | 11.314 |
|  |  |  |  |  |  |  |  |  |  |
|  | *Controls: ratio/median (q1, q3)* | *17m/4f* | *56 (25, 61)* | *24 (12, 30)* | *0.00 (0.00, 0.59)* | *8.774 (6.329, 15.962)* | *10.312 (6.058, 17.497)* | *10.137 (6.254, 12.399)* | *8.287 (5.557,* *15.011)* |
|  |  |  |  |  |  |  |  |  |  |
|  | **Suicide victims** |  |  |  |  |  |  |  |  |
| 22 | Self-poisoning (quetiapine) | m | 59 | 12 | 2.40 | 8.573 | 7.103 | 6.963 | 18.296 |
| 23 | Self-poisoning (alprazolam, mianserin, verapamil) | f | 85 | 24 | <LOQ | 6.592 | 6.994 | 6.337 | 7.043 |
| 24 | Hanging | m | 54 | 24 | <LOQ | 14.384 | 9.102 | 9.079 | 5.067 |
| 25 | Self-poisoning (zopiclone) | m | 58 | 48 | <LOQ | 5.216 | 3.765 | 3.692 | 5.004 |
| 26 | Hanging | m | 22 | 24 | <LOQ | 3.916 | 6.287 | 7.854 | 7.524 |
| 27 | Hanging | m | 62 | 24 | <LOQ | 7.618 | 6.097 | 2.052 | 4.919 |
| 28 | Hanging | m | 38 | 24 | <LOQ | 5.273 | 5.128 | 5.751 | 4.803 |
| 29 | Hanging | f | 38 | 36 | <LOQ | 15.622 | 37.293 | 15.622 | 16.198 |
| 30 | Self-harm by sharp object (stab wound of head) | m | 29 | 24 | 0.80 | 8.896 | 8.016 | 7.311 | 9.722 |
| 31 | Hanging | f | 57 | 24 | <LOQ | 4.259 | 4.652 | 7.387 | 5.528 |
| 32 | Hanging | m | 31 | 12 | <LOQ | 37.787 | 39.931 | 11.709 | 10.204 |
| 33 | Hanging | f | 65 | 24 | <LOQ | 18.285 | 5.033 | 4.601 | 2.118 |
| 34 | Self-poisoning (olanzapine, clomipramine, amlodipine, bisoprolol) | m | 43 | 24 | <LOQ | 5.914 | 7.330 | - | 5.236 |
| 35 | Hanging | m | 47 | 24 | <LOQ | 10.599 | 7.196 | 5.972 | 42.231 |
| 36 | Hanging | m | 53 | 42 | <LOQ | 2.421 | 6.733 | 7.091 | 3.704 |
| 37 | Self-poisoning (chlorprothixen) | m | 42 | 6 | <LOQ | 6.390 | 8.331 | 4.146 | 3.800 |
| 38 | Hanging | m | 28 | 12 | <LOQ | 10.858 | 4.542 | 4.990 | 1.105 |
| 39 | Self-poisoning (morphine) | f | 33 | 48 | 0.35 | 3.499 | 2.140 | 2.401 | 2.318 |
| 40 | Self-poisoning (venlafaxine, trazodone, tramadol) | f | 68 | 24 | <LOQ | 3.499 | 2.140 | 2.401 | 2.318 |
| 41 | Hanging | m | 41 | 72 | <LOQ | 8.586 | 10.523 | 10.664 | 11.186 |
|  |  |  |  |  |  |  |  |  |  |
|  | *Suicide victims: ratio/median (q1, q3)* | *14m/6f* | *45 (35, 58)* | *24 (24, 30)* | *0.00 (0.00, 0.00)* | *7.105 (4.738, 10.729)* | *6.864 (4.843, 8.174)* | *6.337 (4.146, 7.854)* | *5.152 (3.752, 9.963)* |
|  |  |  |  |  |  |  |  |  |  |
|  | **Statistics** |  |  |  |  |  |  |  |  |
|  | test | *χ^2^*-test | *U* | *U* | *U* | *U* | *U* | *U* | *U* |
|  | Characteristic value | *χ^2^*= 0.670 | *Z*= −0.156 | *Z=* −0.587 | *Z*= 0.769 | *Z=* 1.552 | *Z=* 2.231 | *Z*= 2.343 | *Z=* 1.975 |
|  | *P* value | 0.414 | 0.876 | 0.557 | 0.442 | 0.122 | **0.020** | **0.019** | **0.048** |

**Supplementary Table - Continuation** Detailed diagnostic and demographic data, and the values of rRNA relative levels in regions of interest in controls (n=21) and suicide victims (n=20). *Abbreviations:* OFC – orbitofrontal cortex; DLC – dorsolateral prefrontal cortex; f – female; m – male; PMI – postmortem interval; BAC – blood alcohol concentration; LOQ – limit of quantification (BAC = 0.2 g/l); *q1* and *q3* – quartile 1 and 3.

| Case ID | Cause of death | Sex | Age | PMI | BAC | OFC right | OFC left | DLC right | DLC left |
| --- | --- | --- | --- | --- | --- | --- | --- | --- | --- |
|  | **Controls** |  |  |  |  |  |  |  |  |
| 1 | Sudden cardiac death | m | 61 | 24 | <LOQ | 7.984 | 11.043 | 4.272 | 8.545 |
| 2 | Traffic accident (motorcycle rider, injury of subclavian artery and traumatic haemothorax) | m | 25 | 12 | <LOQ | 6.634 | 9.444 | 5.667 | 5.605 |
| 3 | Right ventricular failure | m | 58 | 48 | <LOQ | 5.250 | 3.652 | 3.914 | 3.843 |
| 4 | Sudden cardiac death | m | 63 | 15 | <LOQ | 7.087 | 5.270 | 4.186 | 4.520 |
| 5 | Traffic accident (pedestrian injured in collision with motorcycle, multiple injuries) | m | 24 | 12 | <LOQ | 23.591 | 56.285 | 57.878 | 51.038 |
| 6 | Pulmonary embolism (acute cor pulmonale) | f | 59 | 24 | <LOQ | 5.326 | 4.165 | 5.960 | 2.716 |
| 7 | Traffic accident (car occupant, multiple injuries) | m | 20 | 24 | <LOQ | 5.995 | 4.409 | 6.311 | 6.244 |
| 8 | Traffic accident (car occupant, multiple injuries) | f | 59 | 24 | <LOQ | 4.366 | 5.626 | 9.153 | 7.287 |
| 9 | Traffic accident (pedestrian injured in collision with motorcycle, multiple injuries) | m | 24 | 12 | 0.80 | 7.270 | 5.485 | 8.950 | 4.965 |
| 10 | Sudden cardiac death | m | 45 | 12 | <LOQ | 15.552 | 7.069 | 11.938 | 7.388 |
| 11 | Stabbed (open wounds of thorax) | m | 57 | 10 | 1.70 | 36.403 | 15.740 | 33.449 | 41.968 |
| 12 | Transport accident (pedestrian injured in collision with railway vehicle, injury of cervical spinal cord) | f | 19 | 20 | <LOQ | 20.985 | 8.145 | 19.156 | 9.725 |
| 13 | Stabbed (open wound of thorax) | m | 28 | 7 | 3.15 | 5.319 | 8.737 | 6.990 | 1.917 |
| 14 | Traffic accident (pedestrian injured in collision with car, multiple injuries) | m | 71 | 24 | 0.74 | 15.677 | 35.735 | 9.481 | 10.451 |
| 15 | Traffic accident (car driver, multiple injuries) | m | 32 | 30 | 0.59 | 5.287 | 6.665 | 5.155 | 8.170 |
| 16 | Work-related transport accident (pedestrian injured in collision with railway vehicle, multiple injuries) | m | 56 | 48 | <LOQ | 11.854 | 12.037 | 21.866 | 11.679 |
| 17 | Traffic accident (pedestrian injured in collision with car, multiple injuries) | m | 66 | 24 | <LOQ | 12.037 | 21.866 | 11.679 | 14.491 |
| 18 | Diabetic coma with ketoacidosis | m | 39 | 48 | <LOQ | 8.509 | 14.015 | 14.740 | 12.115 |
| 19 | Sudden cardiac death | m | 64 | 48 | <LOQ | 11.484 | 8.509 | 14.015 | 14.740 |
| 20 | Work-related injury of head | m | 21 | 64 | 1.07 | 17.497 | 10.372 | 9.477 | 7.569 |
| 21 | Accidental fall from building | f | 72 | 15 | <LOQ | 10.523 | 25.386 | 10.535 | 9.477 |
|  |  |  |  |  |  |  |  |  |  |
|  | *Controls: ratio/median (q1, q3)* | *17m/4f* | *56 (25, 61)* | *24 (12, 30)* | *0.00 (0.00, 0.59)* | *8.509 (5.995, 15.552)* | *8.737 (5.626, 14.015)* | *9.477 (5.960, 14.015)* | *8.170 (5.605, 11.679)* |
|  |  |  |  |  |  |  |  |  |  |
|  | **Suicide victims** |  |  |  |  |  |  |  |  |
| 22 | Self-poisoning (quetiapine) | m | 59 | 12 | 2.40 | 7.406 | 19.997 | 6.458 | 8.134 |
| 23 | Self-poisoning (alprazolam, mianserin, verapamil) | f | 85 | 24 | <LOQ | 4.060 | 9.187 | 9.187 | 8.327 |
| 24 | Hanging | m | 54 | 24 | <LOQ | 3.878 | 6.570 | 6.081 | 3.192 |
| 25 | Self-poisoning (zopiclone) | m | 58 | 48 | <LOQ | 10.803 | 5.803 | 3.565 | 4.570 |
| 26 | Hanging | m | 22 | 24 | <LOQ | 5.745 | 6.873 | 7.226 | 5.188 |
| 27 | Hanging | m | 62 | 24 | <LOQ | 4.734 | 4.563 | 12.743 | 7.522 |
| 28 | Hanging | m | 38 | 24 | <LOQ | 6.159 | 5.993 | 7.144 | 4.719 |
| 29 | Hanging | f | 38 | 36 | <LOQ | 10.648 | 9.251 | 2.517 | 31.905 |
| 30 | Self-harm by sharp object (stab wound of head) | m | 29 | 24 | 0.80 | 6.309 | 6.513 | 7.591 | 11.148 |
| 31 | Hanging | f | 57 | 24 | <LOQ | 6.639 | 9.585 | 6.270 | 3.526 |
| 32 | Hanging | m | 31 | 12 | <LOQ | 9.500 | 11.059 | 47.112 | 53.611 |
| 33 | Hanging | f | 65 | 24 | <LOQ | 14.024 | 8.238 | 5.192 | 2.999 |
| 34 | Self-poisoning (olanzapine, clomipramine, amlodipine, bisoprolol) | m | 43 | 24 | <LOQ | 10.767 | 11.106 | 6.217 | 4.727 |
| 35 | Hanging | m | 47 | 24 | <LOQ | 5.722 | 6.303 | 5.909 | 6.331 |
| 36 | Hanging | m | 53 | 42 | <LOQ | 2.139 | 9.108 | 2.995 | 2.942 |
| 37 | Self-poisoning (chlorprothixen) | m | 42 | 6 | <LOQ | 5.872 | 4.993 | 6.210 | 5.214 |
| 38 | Hanging | m | 28 | 12 | <LOQ | 2.064 | 8.575 | 4.538 | 6.363 |
| 39 | Self-poisoning (morphine) | f | 33 | 48 | 0.35 | 2.834 | 2.798 | 2.067 | 2.203 |
| 40 | Self-poisoning (venlafaxine, trazodone, tramadol) | f | 68 | 24 | <LOQ | 2.834 | 2.798 | 2.067 | 2.203 |
| 41 | Hanging | m | 41 | 72 | <LOQ | 8.893 | 10.904 | 9.352 | 21.294 |
|  |  |  |  |  |  |  |  |  |  |
|  | *Suicide victims: ratio/median (q1, q3)* | *14m/6f* | *45 (35, 58)* | *24 (24, 30)* | *0.00 (0.00, 0.00)* | *6.016 (3.969, 9.197)* | *7.555 (5.898,*  *9.418)* | *6.214 (4.052, 7.409)* | *5.201 (3.359, 8.230)* |
|  |  |  |  |  |  |  |  |  |  |
|  | **Statistics** |  |  |  |  |  |  |  |  |
|  | test | *χ^2^*-test | *U* | *U* | *U* | *U* | *U* | *U* | *U* |
|  | Characteristic value | *χ^2^*= 0.670 | *Z*= −0.156 | *Z=* −0.587 | *Z*= 0.769 | *Z=* 2.426 | *Z=* 1.161 | *Z=* 2.295 | *Z=* 1.552 |
|  | *P* value | 0.414 | 0.876 | 0.557 | 0.442 | **0.015** | 0.249 | **0.022** | 0.122 |
